# Identification of TIFY gene family in walnut and analysis of its expression under abiotic stresses

**DOI:** 10.1186/s12864-022-08416-9

**Published:** 2022-03-07

**Authors:** Xuejiao Liu, Feiyan Yu, Guiyan Yang, Xiaoqiang Liu, Shaobing Peng

**Affiliations:** 1 Laboratory of Walnut Research Center, College of Forestry, Northwest A & F University, Yangling, 712100 Shaanxi China; 2 Key Laboratory of Bio-Resource and Eco-Environment of Ministry of Education, College of Life Science, Sichuan University, Chengdu, 610065 Sichuan China; 3 Department of Foreign Languages, Northwest A & F University, Yangling, 712100 Shaanxi China

**Keywords:** Walnut, TIFY gene family, Evolution, Phylogeny, Stress expression analysis

## Abstract

**Background:**

Walnuts (*Juglans regia* L.) are known for their nutrient-rich nuts and are one of the important economic tree species in the world. However, due to global warming and soil salinization, walnuts suffer from various abiotic stresses. TIFY (TIF[F/Y]XG) proteins play an essential role in the growth and development of plants, signal transduction, and stress response in plants. At present, although the TIFY gene family of a number of plants has been identified and studied, how TIFY takes part in stress tolerance remains obscure and many functions of TIFY require further investigation.

**Result:**

In this study, twenty-one TIFY transcription factors were identified in the walnut genome database, and they were divided into four subfamilies (TIFY, JAZ, ZML, and PPD) by bioinformatics analysis. Chromosome location revealed tandem duplication of some genes. Phylogenetic tree analysis showed *JrTIFYs* were closely related to the TIFY gene family of *Arabidopsis thaliana* (*A. thaliana*). qRT-PCR (quantitative real-time PCR) analysis revealed the *TIFY* genes have different expression patterns in ‘Qingxiang’ and ‘Xiangling’ walnut varieties under drought, heat, and salt stress. JAZ subfamily was more expressed in different abiotic stress than other subfamilies. The expressions of *JrTIFY14* under heat and salt stress were significantly higher than those under drought stress. However, the expression of *JrTIFYs* was not significant in ‘Xiangling’.

**Conclusion:**

This study reveals the TIFY gene family plays an important role in walnuts facing abiotic stresses and provides a theoretical basis for walnut breeding.

**Supplementary Information:**

The online version contains supplementary material available at 10.1186/s12864-022-08416-9.

## Background

Walnut (*Juglans regia* L.) is a deciduous tree of the *Juglans* genus of the Juglandacea family, which is the main cultivated economic tree all over the world. Walnut kernels are one of the most important nuts, which are rich in fat, protein, and various vitamins, and walnut is listed as a priority plant of FAO [1]. As global temperatures rise, changes in precipitation systems lead to increased drought stress, resulting in a severe drop in crop yields, especially in Northwest China where walnuts are grown on a large scale [2, 3]. Due to severe drought and soil salinization in this region, during the growth and development of walnut, it is easy to be subjected to drought and high salt stress [4, 5]. Therefore, it is of considerable importance for the walnut to breed heat, drought and salt tolerant cultivars and elucidate the mechanisms of abiotic stress responses [6].

The TIFY (TIF[F/Y]XG) gene family is a novel plant-specific gene family recently identified from *Arabidopsis thaliana* (*A. thaliana*) [7]. This family has a conserved sequence (TIF[F/Y]XG) located in the TIFY domain of about 36 amino acids, which can be divided into four groups: TIFY, JAZ (JASMONATE ZIM-Domain), PPD (PEAPOD), and ZML (ZIM-LIKE), by phylogenetic and structural analysis. Four subfamilies of TIFY gene family have different structures. The characteristic of JAZ proteins is the presence of a C-terminal Jas domain, which interacts with the MYC2 proteins to inhibit the jasmonate acid (JA) signaling pathway [7]. PPD protein contains a Jas domain defecting PY-NLS motif (Jasmonate-associate) and a PPD domain, and ZML protein contains a GATA zinc finger and a CCT motif [8–11]. In TIFY domain, glycine is completely conserved and other hydrophobic amino acids are variable. The secondary predicted structure shows that it forms α-α-β folded structure [10]. Meloto et al. [12] considered that TIFY domain has a non-negligible role in mediating the interaction between members of TIFY domain and other transcription factors (TFs) [13, 14]. Jas domain, also known as CCT-2 domain, contains the characteristic motif of SLx2Fx2KRx2Rx5PY, which can interact with COI1 and other TFs [8, 12, 15–18].

Plants are subject to biological and abiotic stress inevitably and frequently in their life cycles, which negatively affect their growth, development, and productivity. When plants are under stress, some small signal molecules, such as JA, salicylic acid (SA), ethylene (ET), and abscisic acid (ABA), will help to resist these internal and external stresses. Recently, some studies have identified that the TIFY gene family has multiple regulatory roles in cell signal transduction and plant stress response regulation [19]. Although information about *TIFY* genes is lacking in most of the plant species, some studies have shown that TIFY gene family plays a key role in plant stress responses, and JAZ protein is one of the most important components of the jasmonate pathway, which has been identified as a transcriptional inhibitor of JA signal [13, 20]. ZIM is the basis for the formation of homodimers and heterodimers and plays a role in petiole and hypocotyl elongation [18]. Phylogenetic analysis of the GATA gene family in *A. thaliana* revealed that 29 GATA proteins were divided into four subfamilies, three of which encoded ZIM and ZIM-like proteins (ZML proteins). *GATA* genes participate in the light-responsive, circadian-regulated, and tissue-specific expression patterns [18]. PPDI and PPDII participate in leaf growth coordination [15]. Some studies have found that PPDI proteins can coordinate tissue growth, regulate leaf size and inhibit leaf bending, and the PPDII protein interacts with Gemini al2 protein and shell protein promoter in *A. thaliana*. Both PPD proteins are closely related to cell cycle arrest [21–23]. Therefore, TIFY gene family plays a significant role in plant growth, development, and stress tolerance.

Although the TIFY genetic family has been studied in the growth and development of some plant species, and its functions have been identified, such as rice (*Oryza sativa*) [24, 25], soybean (*Glycine max*) [26], tomato (*Lycopersicon esculentum*) [27], and *Salvia miltiorrhiza* (*S. miltiorrhiza*) [11], few studies have been conducted on the stress resistance of *TIFY* genes in *J. regia*. Because of the serious adverse effects of abiotic stresses on the walnut, the study on the TIFY gene family remains necessary. In addition, in our previous research, late-bearing walnuts (‘Qingxiang’) have higher stress resistance than early-bearing walnuts (‘Xiangling’), but the principle is still unknown [28]. In this study, we identified, classified, and made phylogenetic analyses of the TIFY gene family of two walnut species, as well as the levels of *TIFY* genes in walnut expression in three abiotic stresses with a view to providing a theoretical basis for the evolution of the TIFY gene family and future walnut breeding.

## Results

### Genome-wide identification and chromosomal locations of TIFY family genes in *J.regia*

Twenty-one *TIFY* genes were identified from *J. regia* genome by BLAST and named *JrTIFY01*—*JrTIFY21* (Table 1). These genes belonged to four subfamilies, including two members of TIFY subfamily, twelve members of JAZ subfamily, two members of ZML subfamily and two members of PPD subfamily. The molecular weights of TIFY proteins range from 14.46 KDa to 45.68 KDa and amino acids of them range from 126 to 437. The theoretical PIs of the *JrTIFYs* are 5.77 to 9.86. Since *JrTIFY10*, *JrTIFY11*, *JrTIFY13*, and *JrTIFY21* have identical gene IDs and protein characters, they are considered to be the same gene. In the following analysis, *JrTIFY11*, *JrTIFY13*, and *JrTIFY21* are deleted. The results of subcellular location prediction analysis showed that, except for *JrTIFY07* and *JrTIFY16* located in the chloroplast, the remaining 18 *JrTIFYs* were located in the nucleus.

**Table 1:** The *TIFY* genes in *J. regia*

Gene	Accession No	Gene ID	CDS/bp	Number of amino acid/aa	Molecular weight/KDa	Theoretical PI	Subcellular localization
*JrTIFY01*	> XP_018823646.1	LOC108993250	1170	389	41.78	8.69	Nucleus
*JrTIFY02*	> XP_018837866.1	LOC109003977	1159	386	40.77	8.85	Nucleus
*JrTIFY03*	> XP_018845750.1	LOC109009626	1299	432	45.16	8.76	Nucleus
*JrTIFY04*	> XP_018839218.1	LOC109004963	858	285	31.27	9.3	Nucleus
*JrTIFY05*	> XP_018839402.1	LOC109005076	1026	341	37.19	8.54	Nucleus
*JrTIFY06*	> XP_018834762.1	LOC109001793	1314	437	45.68	9.14	Nucleus
*JrTIFY07*	> XP_018835038.1	LOC109001963	798	265	29.21	8.98	Chloroplast
*JrTIFY08*	> XP_018818614.1	LOC108989462	984	327	35.93	9.12	Nucleus
*JrTIFY09*	> XP_018826622.1	LOC108995501	1158	385	40.81	8.62	Nucleus
*JrTIFY10*	> XP_018817541.2	LOC108988673	918	305	32.76	5.77	Nucleus
*JrTIFY11*	> XP_018817541.2	LOC108988673	918	305	32.76	5.77	Nucleus
*JrTIFY12*	> XP_018814737.1	LOC108986549	825	274	29.51	9	Nucleus
*JrTIFY13*	> XP_018817541.2	LOC108988673	918	305	32.76	5.77	Nucleus
*JrTIFY14*	> XP_018840946.1	LOC109006194	1152	383	41.49	9.45	Nucleus
*JrTIFY15*	> XP_018809159.1	LOC108982287	708	235	25.93	8.42	Nucleus
*JrTIFY16*	> XP_018805458.1	LOC108979265	819	272	29.46	9.13	Chloroplast
*JrTIFY17*	> XP_018817541.2	LOC121263054	918	305	32.76	5.77	Nucleus
*JrTIFY18*	> XP_018810390.1	LOC108983260	429	126	14.46	9.86	Nucleus
*JrTIFY19*	> XP_018806456.1	LOC108980095	579	192	20.85	8.79	Nucleus
*JrTIFY20*	> XP_018859193.1	LOC109021098	579	192	21	9.54	Nucleus
*JrTIFY21*	> XP_018817541.2	LOC108988673	918	305	32.76	5.77	Nucleus

**Fig. 1:**
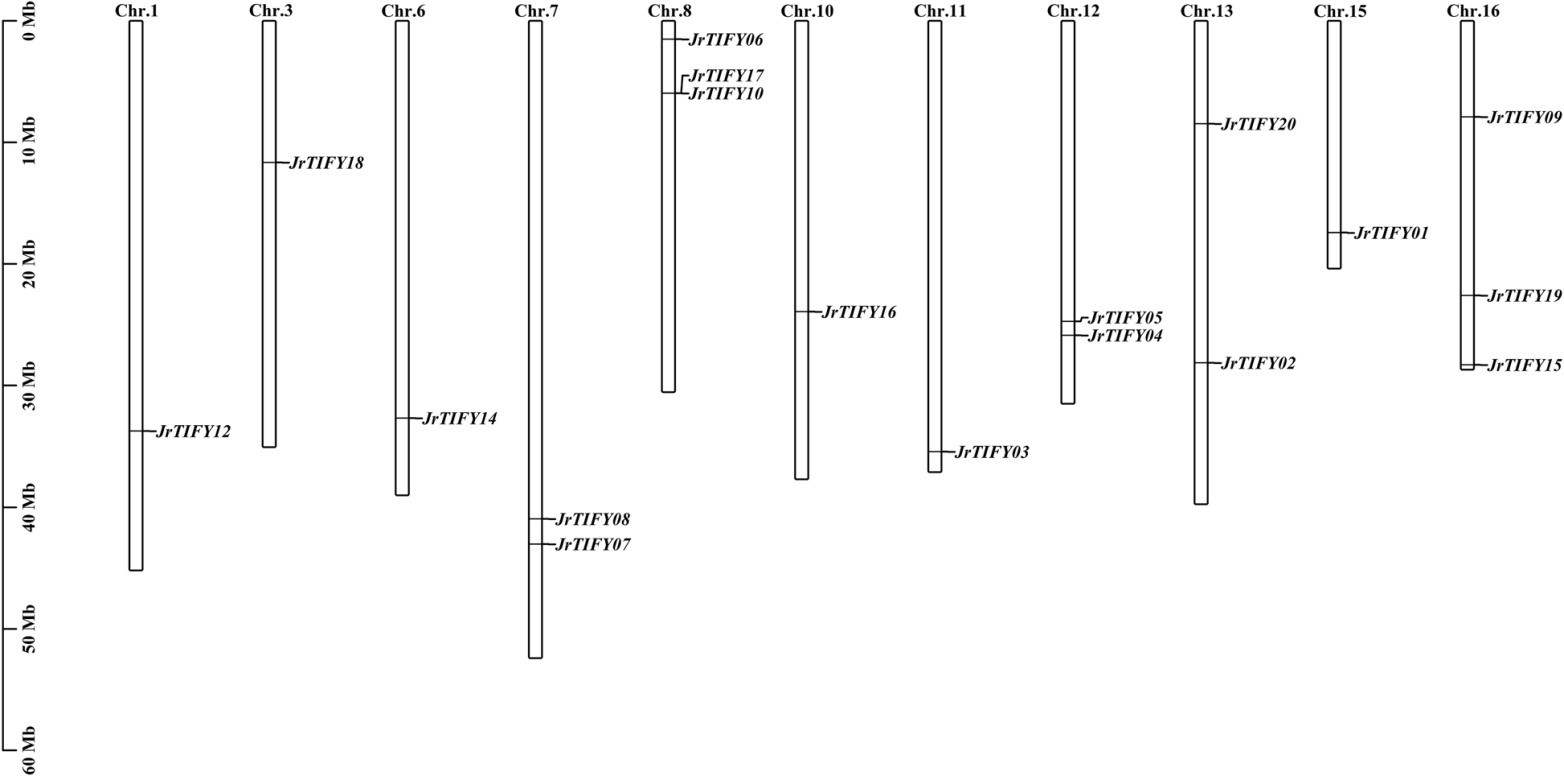
Distribution of the *TIFY*genes on pseudo chromosomes of *J. regia*. The scale on the right is in million bases (Mb)

According to the gene information, chromosome location analysis was carried out for 18 *JrTIFYs* (Fig. 1). A total of 18 *TIFY* genes were located on 11 chromosomes, of which Chr.8 contained three genes (*JrTIFY06*, *JrTIFY10*, and *JrTIFY17*) and Chr.16 contained three genes (*JrTIFY09*, *JrTIFY19*, and *JrTIFY15*). Chr.1, Chr.3, Chr.6, Chr.10, Chr.11, and Chr.15 contained only one *TIFY* gene.

**Fig. 2:**
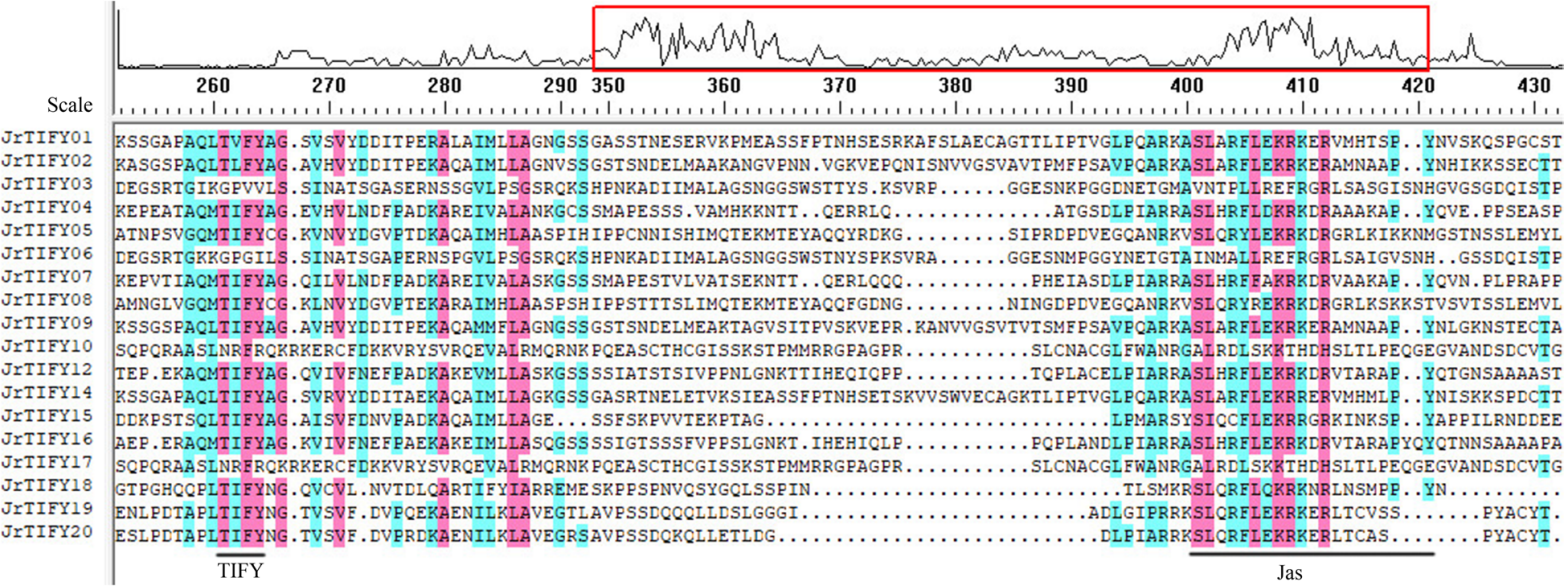
Multiple sequences alignment of *JrTIFYs*. The red box indicates the range of amino acids. The peak of the curve indicates the degree of conservation of amino acids. The scale indicates the number of amino acids. Blue and red indicate conserved amino acids. TIFY: TIFY domain. Jas: Jas domain

### Phylogenetic analysis of walnut TIFY gene family

**Fig. 3:**
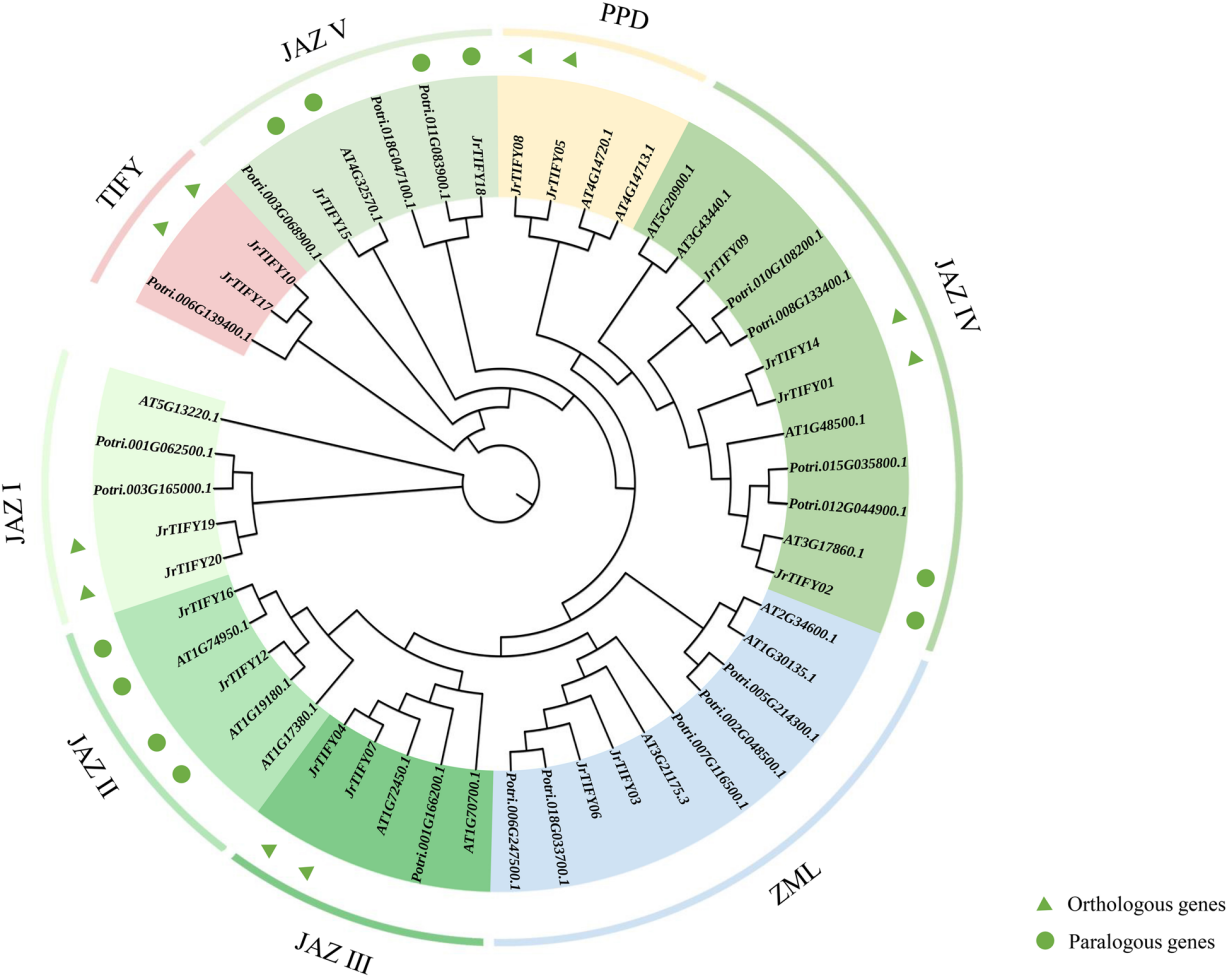
Phylogenetic tree of *TIFY* gene in *P. trichocarpa* (*Potri.*), *A. thaliana* (*AT*) and *J. regia* (*Jr*). Orthologous and paralogous genes were indicated by a triangle and roundness, respectively

Multiple sequence comparisons revealed that most members of the TIFY gene family contain TIFY and Jas domain (Fig. 2). According to genetic differences, a neighbor-joining phylogenetic tree with *TIFY* genes of *J. regia*, *A. thaliana* and *Populus trichocarpa* (*p. trichocarpa*) was built (Fig. 3). A total of 50 genes of *J. regia*, *A. thaliana*, and *p. trichocarpa* were divided into eight branches: proteins containing Jas conservative domain structure were divided into five groups, named groups I-V for JAZ family, including 12 *JrTIFY*s, 11 *AtTIFY*s, and 10 *PtTIFY*s. ZML/ZIM proteins contained 2 *JrTIFY*s, 1 *AtTIFY*s, and 5 *PtTIFY*s. TIFY protein contained 2 *JrTIFY*s and 1 *PtTIFY*s. PPD protein contained 2 *JrTIFY*s and 2 *AtTIFY*s. The JAZ subfamily contained the most *TIFY* genes, and these genes varied widely, showing that the evolution of JAZ subfamily was more extensive in evolutionary history. Of the five branches of the JAZ subfamily, group I, II, III and group V contained two *JrTIFY*s, respectively, group IV contained four *JrTIFY*s. Moreover, the results showed that there were 5 pairs of orthologous genes (*JrTIFY*19 and *JrTIFY20*, *JrTIFY04* and *JrTIFY07*, *JrTIFY14* and *JrTIFY01*, *JrTIFY08* and *JrTIFY05*, *JrTIFY10* and *JrTIFY17*) and 5 pairs of paralogous genes (*JrTIFY*16 and *AT1G74950.1*, *JrTIFY12* and *AT1G19180.1*, *JrTIFY02* and *AT3G17860.1*, *JrTIFY15* and *AT4G32570.1*, *JrTIFY18* and *Potir.011G083900.1*).

### Conservative motif distribution of walnut TIFY gene family

**Fig. 4:**
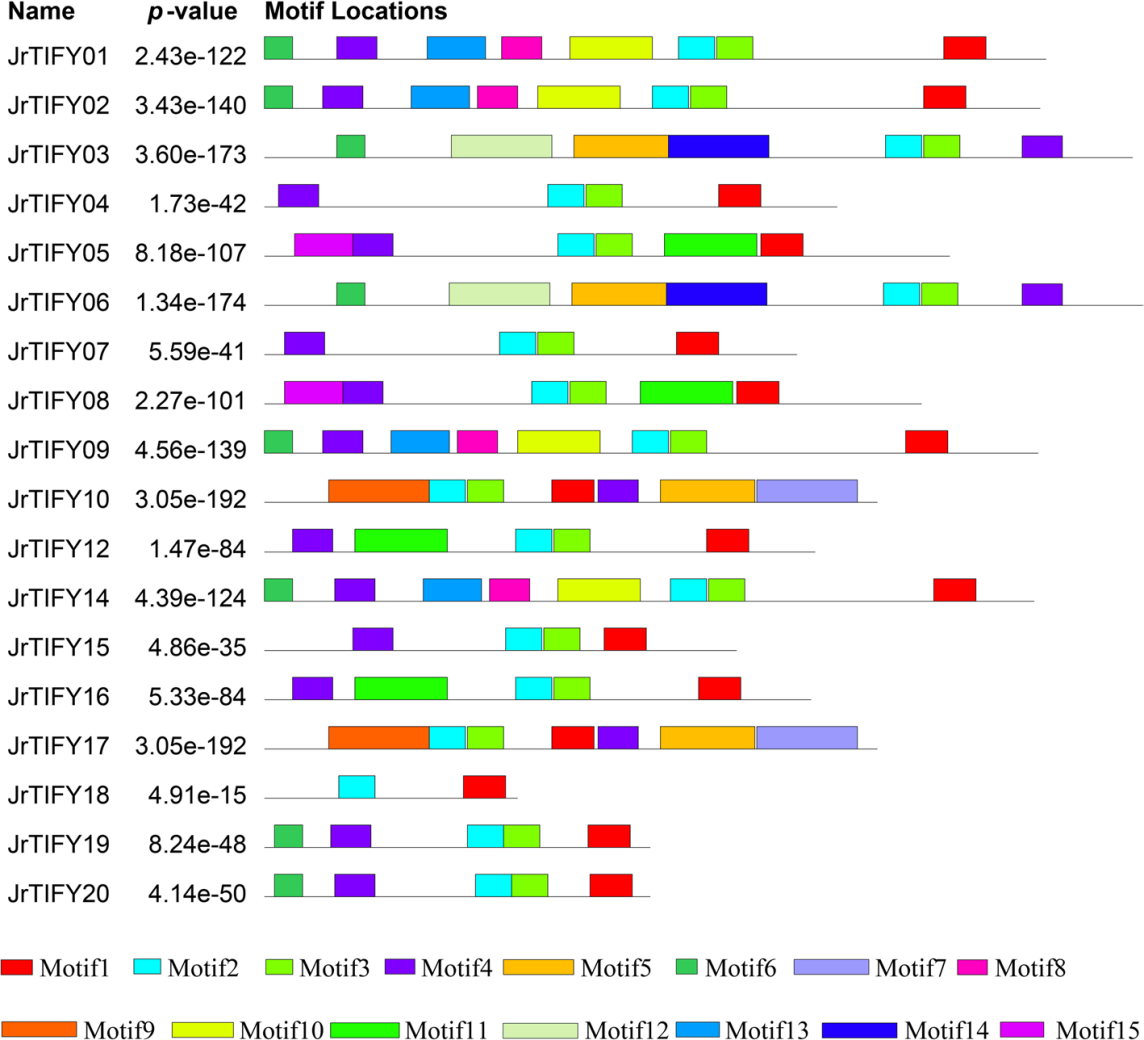
Motifs composition of *JrTIFYs* proteins

**Fig. 5:**
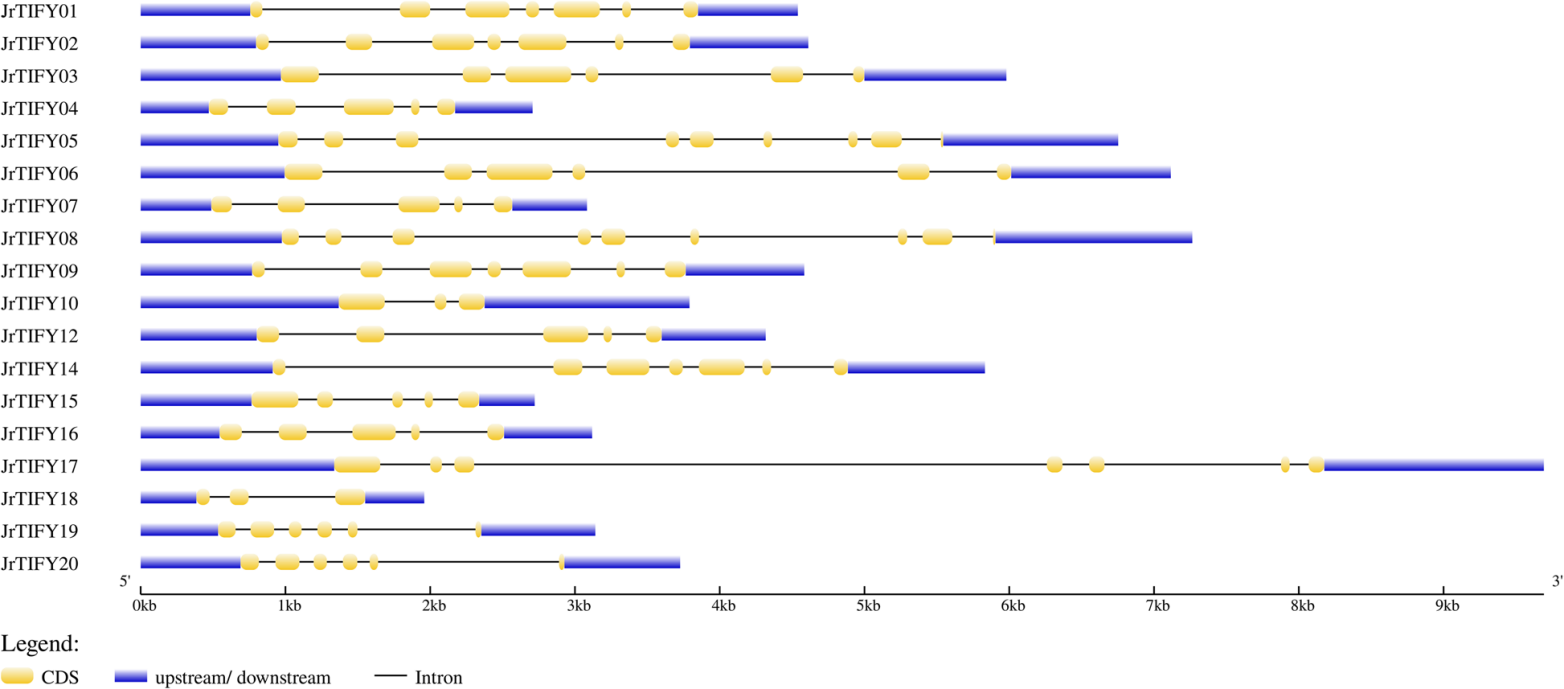
Structure of *JrTIFYs* proteins

According to the conserved regions, MEME motif analysis identified a total of 15 motifs from 18 *JrTIFY*s (Fig. 4 and Table 2). Each *JrTIFY* contained 2–8 motifs. Since TIFY subfamily contains the TIFY domain, motif2 (PATAQLTIFYAGQVHVFD) was annotated as the TIFY domain, and motif1 (LPIARKASLQRFLEKRKERVT) was annotated as Jas domain, which was contained in genes of the JAZ, TIFY and PPD subfamily, but not in the ZIM subfamily. *JrTIFY03* and *JrTIFY06* contained motif12 (NHLEGIPFYGPRSDISGPEISNRIVGSKRSNPDSAFLGSFRDGIINLDHD) and motif14 (WEWPASMNVGPAVKYPPRGGQFGPILHQVPSNRFRDGTAGPSCISQSAAD), none of these in other *JrTIFY*s, which were annotated as GATA zinc-finger and CCT structure. *JrTIFY05* and *JrTIFY08* contained motif15 (ILDKPLNQLTEEDIAQLTREDCRKFLKDK), none of these in other *JrTIFY*s, which were annotated as PPD domain. *JrTIFY10* and *JrTIFY17* have seven same motifs. *JrTIFY01*, *JrTIFY02*, *JrTIFY09*, and *JrTIFY14* contained the same motifs. *JrTIFY18* contained the least domain, only having motif1 and motif2. Moreover, differences in exon–intron structure play an important role in gene evolution. In order to study the diversity of walnut TIFY genes structure, the exon–intron of each TIFY was investigated (Fig. 5). These results showed that most *JrTIFYs* contained more than three exons. *JrTIFY05* and *JrTIFY08* contained the highest number of exons with eight, and *JrTIFY10* and *JrTIFY18* had the fewest number of exons with three. The amino acids motif7, motif9 and motif15 were highly conserved, revealing *JrTIFY*s were highly conservative (Fig. S1).

**Table 2:** Motif sequences identified by MEME tool

Motif	Number of amino acids	Best possible match
Motif1	21	LPIARKASLQRFLEKRKERVT
Motif2	18	PATAQLTIFYAGQVHVFD
Motif3	18	VPPDKAQAIMLLAGKGSS
Motif4	20	GMQRSFRNKCSAJSQYNKEK
Motif5	47	RWGAGGESGRDDNPDEVSFTHCPJRPKSAPLVRRPPAGPRSDANVSG
Motif6	14	MERDFLGLSKKTDS
Motif7	50	FWANRGALRDLSKKTHDHSLTLPEQGEGVANDSDCVTGIHTNNNLVTYSN
Motif8	20	YPPQQHDAHSVHRPHDVKIF
Motif9	50	IDNSLIRYEAHSIDDAAGSVGGVVDDVTADAVYSHGGSDGGASEMVIQRH
Motif10	41	GGTPFFKNHFATGQNLVGSTIKPPPLGGIPPVAPHPSTPSI
Motif11	46	CNJZGNGDKDHLIPPAPTMSLFPQTEKLTDYAQQNVDAGQNPKPPD
Motif12	50	NHLEGIPFYGPRSDISGPEISNRIVGSKRSNPDSAFLGSFRDGIINLDHD
Motif13	29	TADVFDTNKKPYSGEIQKNLNHDKQGGTH
Motif14	50	WEWPASMNVGPAVKYPPRGGQFGPILHQVPSNRFRDGTAGPSCISQSAAD
Motif15	29	ILDKPLNQLTEEDIAQLTREDCRKFLKDK

### Cis-acting element analysis of walnut TIFY gene family

**Fig. 6:**
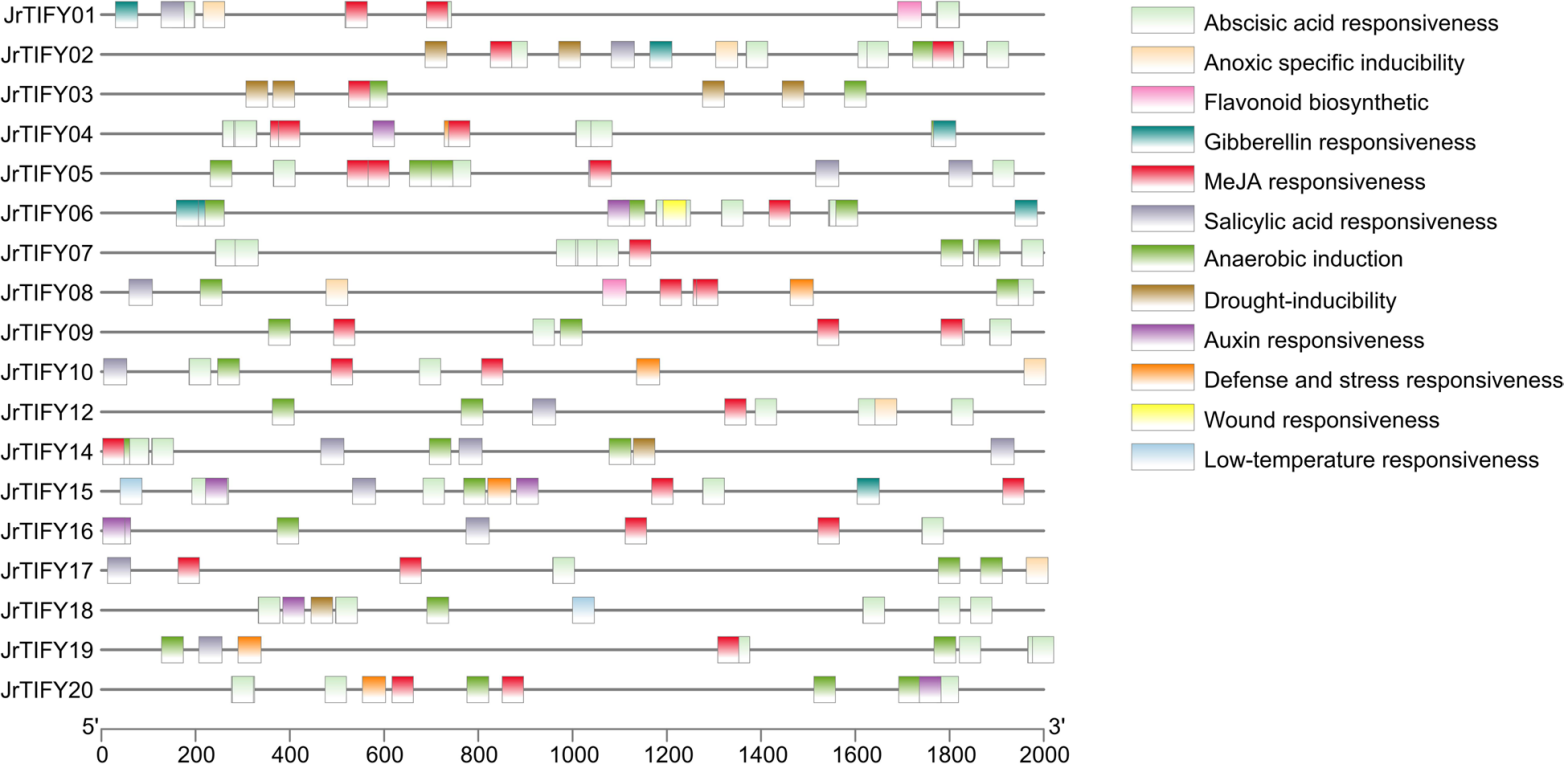
Promoter element of *JrTIFYs* genes

In order to study the potential biological functions of the *JrTIFYs*, the genomic sequence of 2000 bp upstream of the *JrTIFYs* was extracted as a hypothetical promoter sequence for cis-acting element analysis (Fig. 6). The results showed that there were four types of cis-elements in the 18 members, which were plant photoreaction elements, hormone response elements, plant growth and development elements, and stress response elements (Fig. S3). In addition to *JrTIFY03*, other *JrTIFYs* all contained ABA response elements, which were related to the opening and closing of stomata, osmotic stress and stress resistance [29]. In addition to *JrTIFY18*, other *JrTIFYs* all contained MeJA response elements, which stimulated the expression of defensive plant genes [30]. More than half of the genes contained gibberellin response elements, SA response elements and auxin response elements. In addition, some genes contained elements related to stress response such as drought, anaerobic induction, low temperature, and wound response. *JrTIFY01* and *JrTIFY08* contained flavonoid synthesis elements, which were also closely related to stress response [31].

### Expression characteristics of the TIFY gene family under abiotic stresses

**Fig. 7:**
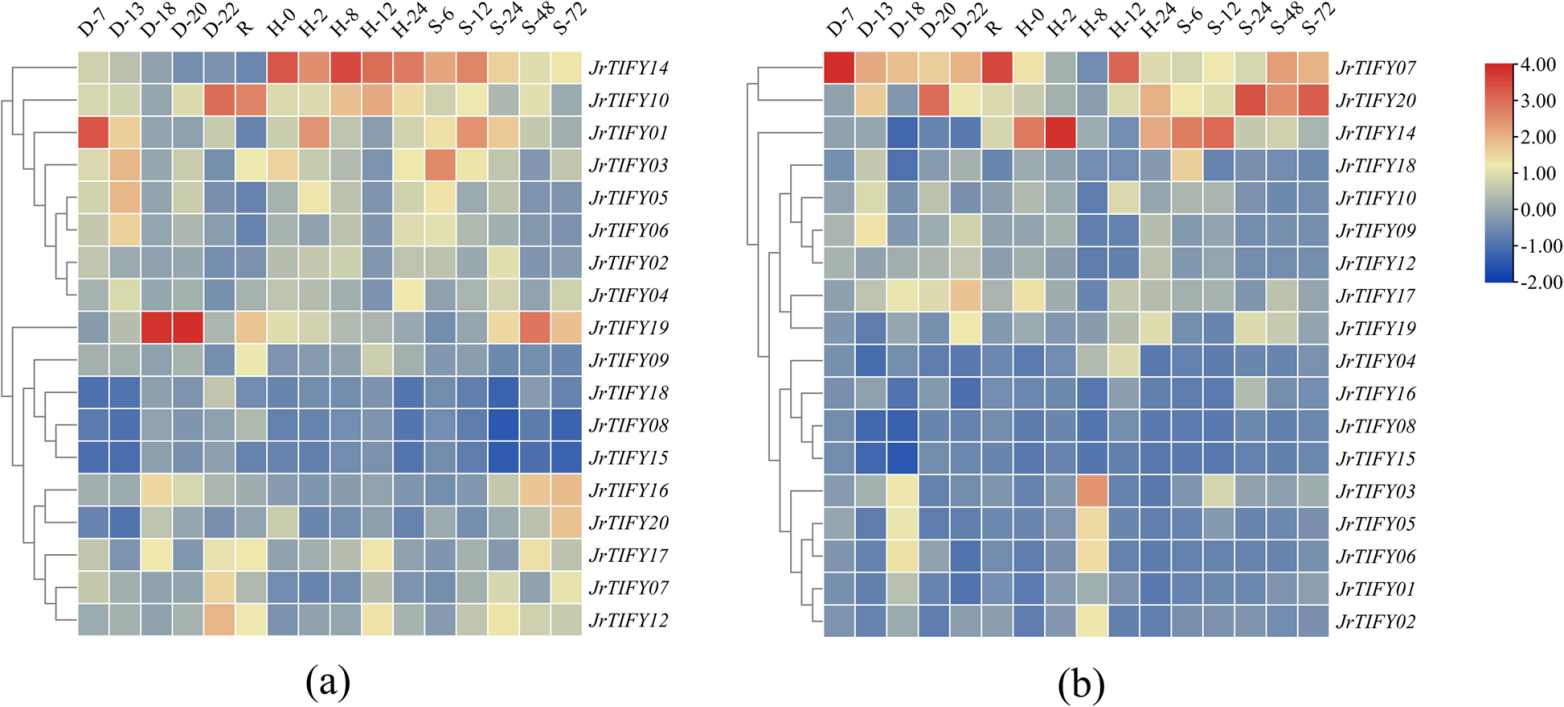
Stress expression profile of *JrTIFY*s. D-7 / D-13 / D-18 / D-20 / D-22: 7, 13, 18, 20, 22 h after drought treatment. R: rehydration after drought treatment. H-0 / H-2 / H-8 / H-12 / H-24: 0, 2, 8, 12 and 24 h after heat treatment. S-6 / S-12 / S-24 / S-48 / S-72: 6, 12, 24, 48 and 72 h after salt treatment. **a** Three abiotic stresses of ‘Qingxiang’; **b** Three abiotic stresses of ‘Xiangling’

The expression characteristics of eighteen *JrTIFYs* in leaves of ‘Qingxiang’ and ‘Xiangling’ walnut varieties under drought, heat, and salt stresses were shown in Fig. 7.

For ‘Qingxiang’ (Fig. 7a). Under three stresses, the expression of some *JrTIFY*s was high, such as *JrTIFY10*, *JrTIFY01*, *JrTIFY03*, and *JrTIFY19*, and the expression of *JrTIFY14* was higher in heat stress and salt stress than in drought stress. However, the expression of some *JrTIFY*s was low, such as *JrTIFY18*, *JrTIFY08*, and *JrTIFY15*. Under drought stress, *JrTIFY10*, *JrTIFY01*, *JrTIFY03*, *JrTIFY05*, and *JrTIFY06* reached the trough value in 18 d (D-18). While *JrTIFY19* showed the high expression in 18 d (D-18) and 20 d (D-20). *JrTIFY10* showed the highest expression in 22 d (D-22), and its expression decreased after rehydration (R). The expression of *JrTIFY01* was the highest at 7 d (D-7). Under heat stress, *JrTIFY08, JrTIFY15,* and *JrTIFY18* showed low expressions throughout the whole process of heat stress, while *JrTIFY*14 showed a high expression throughout the whole process with the highest content at 8 h (H-8). The expression of *JrTIFY01* was the highest at 2 h (H-2). Under salt stress, the expressions of *JrTIFY09*, *JrTIFY18*, *JrTIFY08*, and *JrTIFY15* were low, while the expressions of *JrTIFY14*, *JrTIFY10*, and *JrTIFY01* reached peak value at 24 h (S-24). *JrTIFY19* was particularly prominent with a increase in content from 6 to 24 h, but decreased at 72 h (S-72).

For ‘Xiangling’ (Fig. 7b). Under drought, heat, and salt stress, the expression of *JrTIFY07* and *JrTIFY20* was high, and the expression of *JrTIFY14* was similar to that in ‘Qingxiang’, but the overall expressions of most *JrTIFY*s were very low with almost no expression of *JrTIFY08* and *JrTIFY15*. Under drought stress, the expression level of *JrTIFY07* gradually decreased with time (D-7 ~ D-22), but increased after rehydration (R). Under heat stress, *JrTIFY14* showed the highest expression in 2 h (H-2), and *JrTIFY03*, *JrTIFY05*, *JrTIFY06*, *JrTIFY01*, and *JrTIFY02* reached the peak in 8 h (H-8). Under salt stress, the expressions of *JrTIFY07* and *JrTIFY20* showed an increasing trend over time, but *JrTIFY07* showed a decreasing trend (S-6 ~ S-72). It is worth mentioning that *JrTIFY07* and *JrTIFY20* were highly expressed in ‘Xiangling’, but low in ‘Qingxiang’, while *JrTIFY01* and *JrTIFY03* were opposite under three stresses.

## Discussion

### The walnut *TIFY* genes display diverse characters

With the development of genomics, more and more plant genomes have been analyzed and the TIFY gene family as a specific TF has been identified in more plants. A total of 18 *JrTIFY*s were identified, and phylogenetic trees were established with sixteen genes from *A. thaliana* and sixteen genes from *P. trichocarpa* (Fig. 3). There were four paralogous gene *JrTIFY*s between the walnut and *A. thaliana* (*JrTIFY16* and *AT1G74950.1*, *JrTIFY12* and *AT1G19180.1*, *JrTIFY02* and *AT3G17860.1*, *JrTIFY15* and *AT4G32570.1*), indicating the relationship between the two plant species was closely related during the evolution [10]. Moreover, there was a pair of *JrTIFY*s (*JrTIFY18* and *Potri.011G083900.1*) with similarity in walnut and *P. trichocarpa*. *JrTIFY03* and *JrTIFY06* belonged to the ZML subfamily and closely related to the ZML subfamily of *AT3G21175.3* (*AtZML01*) and *Potri.007G116500.1* (*PtZML02*). Wen et al. [32] think that two genes of the ZML subfamily (*CsTIFY3* and *CsTIFY12*) also have a very high similarity, and Zhao et al. [33] find that several pairs of homologous genes of the ZML subfamily have a very high similarity, indicating that the ZML subfamily may have evolved without variation.

The online tool MEME was used to search for fifteen conservative structures of JrTIFY protein (Fig. 4). Among them, motif1 was predicted to be Jas domain, motif2 to be TIFY structure, and motif12 and motif14 to be GATA zinc-finger structure and CCT structure. *JrTIFY19* and *JrTIFY20* were the same motifs and the located in the closest phylogenetic branches, which belonged to the JAZ subfamily. In Zhao's [33] studies, *PviTIFFY10* and *PviTIFFY11* are located in the closest phylogenetic branches, containing the same motifs, namely N-terminal, TIFY, Jas, belong to the JAZ subfamily. Ye et al. [25] think that just *OsTIFY10* and *OsTIFY11* of the JAZ subfamily in rice have NT motif at N-terminal. These results indicated that the special structure of genes during the evolution of plant species will make the species unique. Moreover, the chromosome location showed that *JrTIFY10* and *JrTIFY17* were at the same position on the same chromosome, indicating that there may be redundancy or tandem duplications, which was consistent throughout the study of Zhang et al. [34]. The study of Zhang et al. shows that *VvJAZ5*, *VvJAZ6*, *VvJAZ7* and *VvJAZ8* are tandem duplications in grapes, which may be the main reason for the expansion of the plant genes’ family.

Among the JAZ subfamily, twelve *JrTIFY*s contained Jas domain, which was the most numerous subfamily, similar to the TIFY gene family of other species such as rice [24, 25], *S. miltiorrhiza* [11] and soybean [26], speculating that the JAZ subfamily may have greater potential in regulating plant growth and development [10]. Previous studies have shown [7, 35] that the JAZ subfamily is an important regulatory factor that controls the initiation of JA by responding to JA stimulation. The JAZ protein usually acts as an inhibitor in the JA signaling pathway, releasing targeted TFs [36]. Through the analysis of promoter elements, we found that almost *JrTIFY*s contained MeJA response elementsm. The research of Lotfi et al. [37] shows that JA is the key hormone for sensitive genotypes to resist stress. It was speculated that TIFY gene family may participate in walnut resistance through JA pathway. Meanwhile, the JAZ subfamily also plays an important role in ABA signaling pathway regulation and plant physiological processes, such as photosynthesis [11, 32, 38]. The study of Zhao et al. [26] suggests that the JAZ subfamily in soybean may be involved in JA signaling pathway to resist saline alkali stress and *GsJAZ*2 overexpression promotes soybean growth in alkaline environment. The JAZ subfamily of *S. miltiorrhiza* negatively regulates the biosynthesis of salvianolic acid and tanshinone [11]. The research of Zhao et al. [33] shows that the *PviTIFY10* of the JAZ subfamily could be induced by ABA in switchgrass (*Panicum virgatum*). Therefore, it can be speculated that the JAZ subfamily has a certain role in different physiological processes in the process of walnut growth.

## *JrTIFYs* expression patterns to abiotic stress implied multiple roles

Under the three stresses, most *JrTIFY*s showed a higher expression level of ‘Qingxiang’ than ‘Xiangling’ (Fig. 7), showing that ‘Qingxiang’ may be more resistant than ‘Xiangling’, which is also in line with previous research [28]. The study of An et al. [39] also prove that overexpression of apple (*Malus domestica*) *MdJAZ2* improves the resistance of *A. thaliana* to NaCl and drought treatments. Similarly, the overexpression of *OsTIFY11a*/*OsJAZ9* in rice significantly enhanced tolerance to salt and dehydration stress [35]. Gene expression profiles of *JrTIFY*s were different under drought, heat, and salt stress. For example, the response of *JrTIFY14* to salt stress and heat stress was stronger than that of drought stress, indicating that *JrTIFY14* may have a negative regulatory effect on drought stress. Xie et al. [40] carries out that all homologous genes of *TaJAZ3* are highly activated by heat, but are significantly inhibited by drought in wheat (*Triticum aestivum*). Similarly, He et al. [41] think *TIFY11b*, *TIFY10a*, *TIFY3*, *TIFY5a/b*, and *TIFY6* of JAZ subfamily are induced by cold acclimation but not by the chilling treatment in *Brassica napus*. The expression profiles of the same *JrTIFY* were also different under the same stress of different walnut varieties. The expression of *JrTIFY01* in ‘Qingxiang’ first decreased and then increased under drought stress, while the expression level first increased and then decreased in ‘Xiangling’. In addition, under the same stress, the *JrTIFYs* had different stress time to reach the maximum expression. For example, under salt stress, the expression level of *JrTIFY20* reached its peak in ‘Qingxiang’ in 72 h, and reached its peak in ‘Xiangling’ in 24 h. This phenomenon was consistent with Wang's [42] research, indicating that different genes had the different response time to stress.

The members of *JrTIFYs* had different response mechanisms under different stresses of the same species or under the same stress of different species. The expression of *JrTIFY10, JrTIFY01*, and *JrTIFY03* was significantly different in ‘Qingxiang’ and ‘Xiangling’, they were more significant in ‘Qingxiang’, but hardly expressed in ‘Xiangling’, but *JrTIFY07* and *JrTIFY20* were opposite, speculating that the resistance of early and late walnuts to abiotic stresses may be related to these genes [43]. Under the same stress, the genes that came from the JAZ subfamily had the most significant expression levels in varieties. For ‘Qingxiang’, the expression of *JrTIFY19* had the greatest change under salt stress, while for ‘Xiangling’, the expression of *JrTIFY20* had the greatest change. Liu et al. [44] believes that *TIFY* genes have different responses to different hormone treatments, and the expressions of genes belonging to the JAZ subfamily increases after MeJA treatment, but decreases after SA/ET treatment, revealing that JAZ plays an important role in the response of plants to abiotic stress.

In the four subfamilies of the TIFY gene family, the expression amount of the JAZ subfamily changed more significantly under different abiotic stresses. The study of Heidari et al. shows that the JAZ subfamily responds more strongly to abiotic stress than other subfamily in maize (*Zea mays*) and tomato [19]. Our research results led to similar conclusions, for instance, *JrTIFY14*, belonging to the fourth branch of the JAZ subfamily, was highly expressed under heat and salt stress, indicating that *JrTIFY14* may have a certain effect on walnuts in response to abiotic stress, which was consistent with Wang's [10] research results. Wang believs that the JAZ subfamily of poplar (*populus*) has a high expression level in different tissues under different stresses, and *PtJAZ8* is significantly expressed in all tissues. *PtJAZ6*, *PtJAZ7*, and *PtJAZ12* were highly expressed in the phloem, xylem, and leaf tissues. Zhang et al. [38] believes that JA induces all *CsJAZs* in tea plants, and these genes show different responses to mechanical injury and bacterial infection. However, under the three stresses of walnut, the basically unexpressed *JrTIFY15* also belonged to the JAZ subfamily, while in Wang's study [8], *PtJAZ1* shows low expression in different tissues of poplar, suggesting that different *JAZ* genes possess distinct functions, and JAZ subfamily may play an obvious role in resistance to abiotic stresses in the plant.

## Conclusions

In this study, a total of 18 *JrTIFY*s were identified and their chromosomal distribution, conserved domain structure, and phylogenetic tree were analyzed. The study concluded that *JrTIFY*s were highly conserved and were closely related to the TIFY gene family of *A*. *thaliana*. Under the stress of drought, heat, and salt, the expressions of different genes were various, in particular, the *JrTIFY14* of JAZ subfamily positive feedback regulated heat and salt stress in both varieties, but negative feedback regulated drought stress. JAZ subfamily played an important role in plant growth and development. However, different walnuts had different responses to abiotic stress. The expression of *JrTIFY*s in ‘Qingxiang’ and ‘Xiangling’ revealed that *JrTIFY*s may play a key regulatory role in the abiotic stress on walnuts. This study provides a theoretical basis for further study on the regulatory mechanism of TIFY gene family and the genetic breeding of walnut.

## Materials and methods

### Plant materials and treatments

#### Seedling preparation

Two walnut varieties ‘Qingxiang’ (late-bearing walnuts) and ‘Xiangling’ (early-bearing walnuts), two-year-old seedlings, were treated with drought, salt, and heat stress. Under the conditions of 22 ± 2℃, 70 ± 5% relative humidity and 14 h light/10 h dark, new branches scions of walnut grafted into the walnut rootstocks (*J. regia*) which were planted in the mixed substrate of turf peat and sand (2:1 v/v) in the greenhouse located in Northwest A&F University (Yangling, Shaanxi province, China). Walnut seedlings with a height of 55–65 cm had similar biomass, and walnut leaves were collected as experimental samples, which were frozen in liquid nitrogen immediately and stored in -80℃ refrigerator for further experiments [28].

#### Drought treatment

Firstly, fifteen ‘Qingxiang’ and fifteen ‘Xiangling’ walnuts were transplanted into the prepared flowerpots with the same conditions, and the soil moisture content of each pot was maintained at 60% for seven days. When the soil water content reached 60% (7 d), 40% (13 d), 20% (18 d), 10% (20 d), and 5% (22 d) without watering, the leaves of nine plants of each variety were collected for three repeated groups. Three plants of the two varieties continued to be re-watered after 22 d to make the soil moisture content reach 60% and kept for 3 days, collecting leaves. The remaining three plants of the two varieties were watered as control [28, 45].

#### Heat treatment

Twelve ‘Qingxiang’ and twelve ‘Xiangling’ walnut seedlings were subjected to heat stress at 42℃ for 2 h. Leaves were collected respectively for three repeated groups at 0, 2, 8, 12, and 24 h after treatment. In addition, three seedlings of each walnut variety were cultured at 25℃ as control [28].

#### Salt treatment

A total of twenty-four plants (twelve plants of each variety) were treated with ½ Hoagland with 300 mM NaCl. The leaves were collected at 6, 12, 24, 48, and 72 h after treatment respectively for three repeated groups. In addition, three seedlings of each walnut variety were watered with NaCl-free ½ Hoagland solution as control [28, 46].

### Identification and chromosomal locations of the walnut TIFY gene family

The conserved domain information of *TIFY* genome was downloaded from Pfam website (http://pfam.sanger.ac.uk), and the chromosome position of TIFY gene family in the walnut genome (*J. regia*) was obtained on NCBI website (https://doi.org/10.1093/gigascience/giaa050) [47]*.* The *TIFY* genes sequence information of walnut was downloaded from iTAK website (http://itak.feilab.net/cgi-bin/itak/db_family_gene_list.cgi?acc=Tify&plant=51240). The TIFY gene family of walnut, *A. thaliana* and *P. trichocarpa* was searched by blast on NCBI website, and the coding regions’ gene sequences of *TIFY*s were obtained by UltraEdit software [48, 49]. The chromosome location, sequence ID, protein sequence, amino acid number, protein molecular weight (kDa), and protein isoelectric point (PI) were downloaded from ExPASY website (http://web.expasy.org/protparam/). The predictive analysis of the subcellular localization of walnut TIFY protein was performed by using the WoLF PSORT (https://www.genscript.com/wolf-psort.html) [50].

### Multiple alignments and phylogenetic analyses

Phylogenetic reconstruction is an important part of genomics. DANMAN software was used for multiple comparison of walnut TIFY protein. Clustal × 2.1 software was used to multi-aligned amino acid sequences of TIFY proteins in walnut [51]. The phylogenetic tree was established by using MEGA6.0 software with a neighborhood connection method for the protein sequences of TIFY in walnut, *A. thaliana* and *p. trichocarpa* (bootstrap = 1000) [52]. The amino acid sequence with FASTA format was input into the online tool MEME (Multiple Em for Motif Elicitation) (http://meme-suite.org/tools/meme) to predict the structures, and the conservative motif searched was 15, and the others are default values [53]. The 2000 bp upstream of the start codon of the walnut TIFY gene family was extracted and uploaded to PlantCARE (http://bioinformatics.psb.ugent.be/webtools/plantcare/html/) by using TBtools software to predict and analyze the cis-acting elements in the promoter region [50].

### qRT-PCR analysis

Total RNA was extracted from walnut leaves of ‘Qingxiang’ and ‘Xiangling’ varieties by CTAB method [54]. Because four of the *TIFY* genes (*JrTIFY10*, *JrTIFY11*, *JrTIFY13*, and *JrTIFY21*) are so similar that the primers designed are the same, only one of the *TIFY* genes (*JrTIFY10*) was used in the experiment. Gene-specific primers were given in Table S1. A total of 36 *TIFY* genes of two varieties were amplified respectively by PCR. Then qRT-PCR (quantitative real-time PCR) was used to measure 20 μL reaction mixture with TB Green™ Premix Ex TaqTMII (TaKaRa, Xi’an, Shaanxi, China) on Bio-Rad CFX ManagerTM Software3.1 in triplicate. The reaction procedure was as follows: 95℃, 30 s pre denaturation; 95℃, 5 s denaturation; annealing to 58℃, 30 s, then heating to 72℃ 30 s, 40 cycles, the last 95℃, 10 s. The qRT-PCR for each gene had three replicates [28]. The *18S-RNA* gene was used as a control [55]. The data was analyzed and processed. The relative expression was calculated as 2^−△△Ct^ [56]. The experimental details were shown in Table S2.

## Supplementary Information

**Additional file 1. **

**Additional file 2. **

**Additional file 3. **

**Additional file 4. **

**Additional file 5. **

## Data Availability

All data generated or analyzed during this study are included in this published article [and its supplementary information files]. The domain architecture of the *TIFY* genes was obtained from Pfam database (http://pfam.sanger.ac.uk). The chromosomal locations in the walnut genome (*J. regia*) were downloaded from NCBI database (https://doi.org/10.1093/gigascience/giaa050). The walnut *TIFY* gene sequences were downloaded from iTAK (http://itak.feilab.net/cgi-bin/itak/index.cgi) database. ExPASy proteomics server (https://web.expasy.org/protparam/) was used to estimate the amino acid number, the molecular weight and the theoretical isoelectric point (PI). The MEME tools (http://meme-suite.org/tools/meme) was chosen to analyze the conservative motifs. The WoLF PSORT (https://www.genscript.com/wolf-psort.html) was used to analyze subcellular localization. The PlantCARE (http://bioinformatics.psb.ugent.be/webtools/plantcare/html/) was used to predict the cis-acting elements in the promoter region.

## Abbreviations

TIFY: TIF[F/Y]XG
JAZ: Jasmonate ZIM-Domain
PPD: PEAPOD proteins
ZML: ZIM-LIKE proteins
ZIM: Zinc-finger protein expressed in Inflorescence Meristem
qRT-PCR: Quantitative real time polymerase chain reaction
DBD: DNA-binding domain
FAO: The Food and Agriculture Organization
JA: Jasmonate acid
GATA: (A/T)GATA(A/G) sequence
TFs: Transcription factors
COI1: CORONATINE INSENSITIVE 1
SA: Salicylic acid
ET: Ethylene
ABA: Abscisic acid
