# Supplementary material for: Identification of TIFY gene family in walnut and analysis of its expression under abiotic stresses
Source: BMC Genomics. 2022 Mar 7;23:190. doi: 10.1186/s12864-022-08416-9 (PMC8903722; doi:10.1186/s12864-022-08416-9)
Supplement: Supplementary file 1 — Additional file 1. [file 12864_2022_8416_MOESM1_ESM.docx]

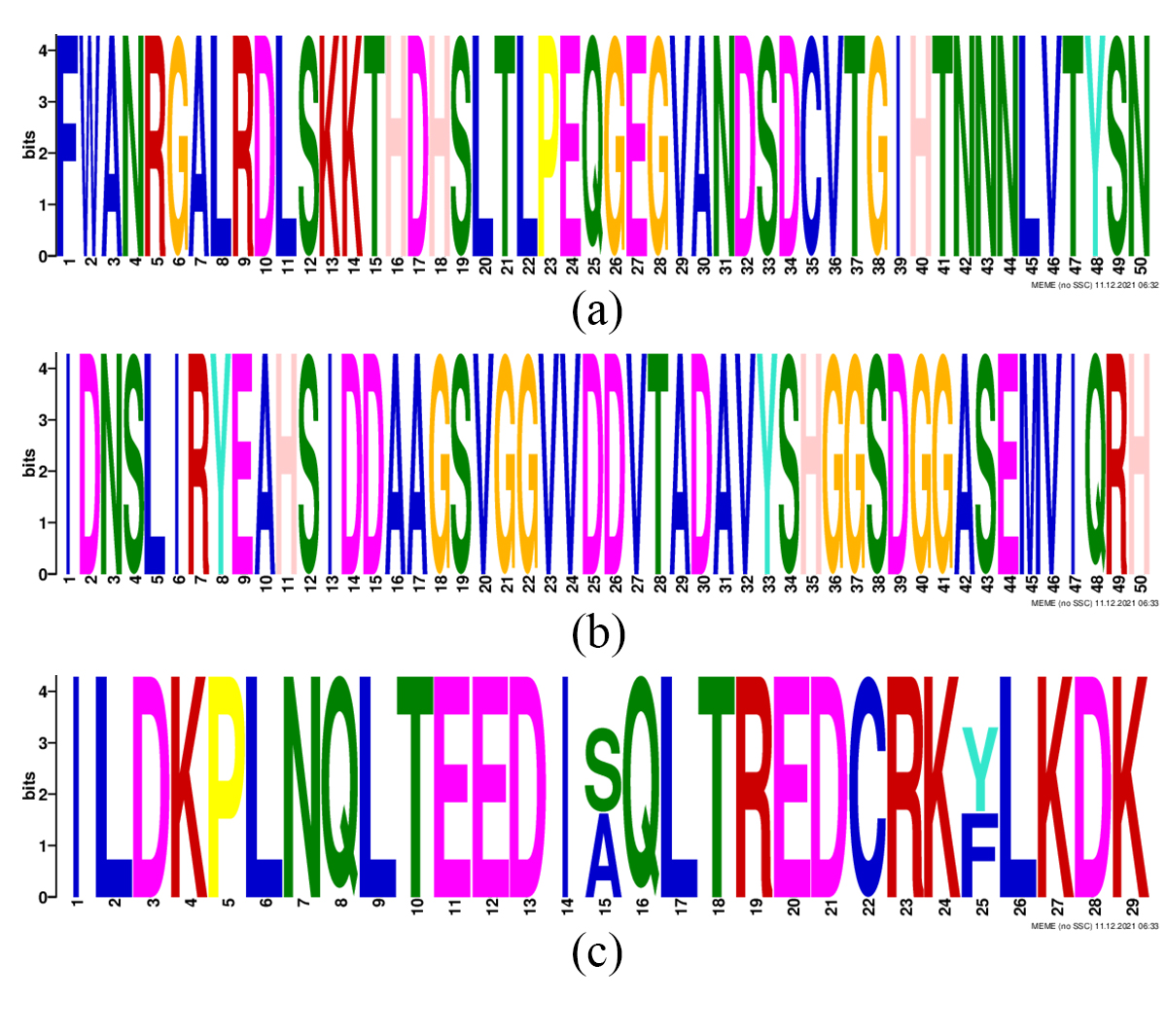


**Supplemental Figure 1. Highly conserved motifs of JrTIFYs.** (a) The Weblogo of motif7. (b) The Weblogo of motif9. (c) The Weblogo of motif15.
