# Supplementary material for: Identification of TIFY gene family in walnut and analysis of its expression under abiotic stresses
Source: BMC Genomics. 2022 Mar 7;23:190. doi: 10.1186/s12864-022-08416-9 (PMC8903722; doi:10.1186/s12864-022-08416-9)
Supplement: Supplementary file 2 — Additional file 2. [file 12864_2022_8416_MOESM2_ESM.docx]

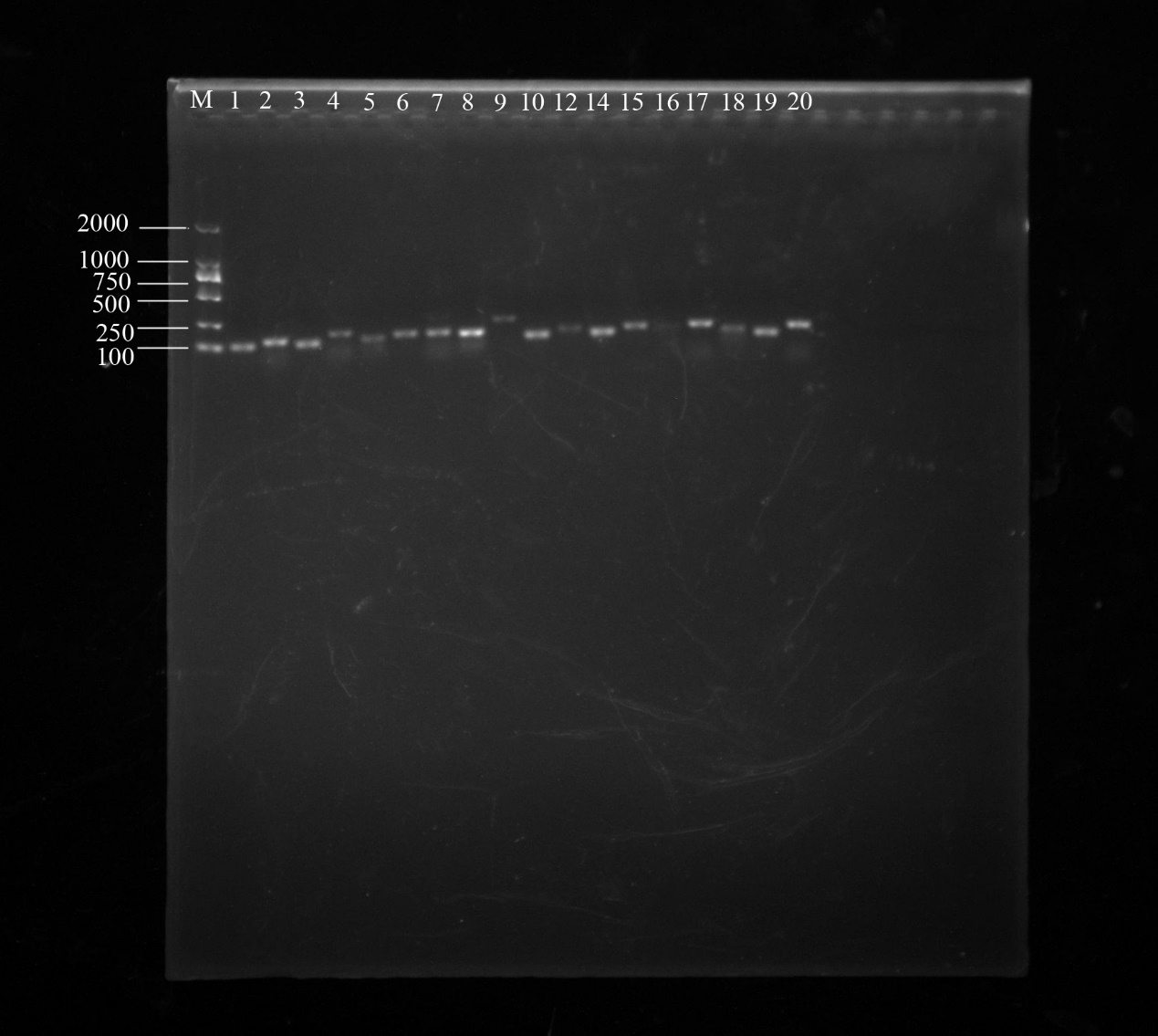


(A)


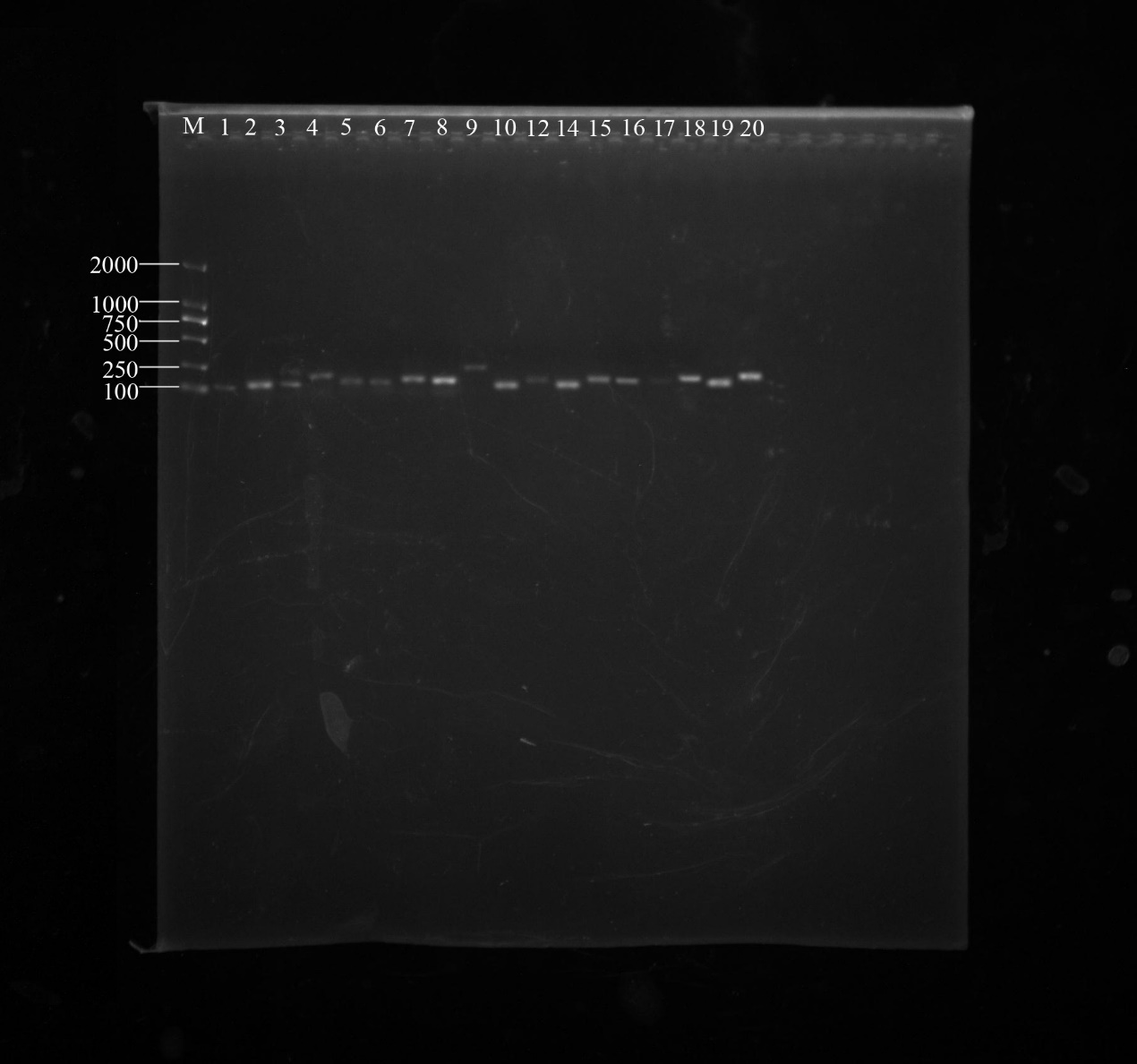


(B)

**Supplemental Figure 2. PCR analysis of *JrTIFY* in ‘Qingxiang’ and ‘Xianging’.** M: DL Marker2000. (A) 01-20: *JrTIFY01*-*JrTIFY20* in ‘Qingxiang’. (B) 01-20: *JrTIFY01*-*JrTIFY20* in ‘Xiangling’.
