# Supplementary material for: Identification of TIFY gene family in walnut and analysis of its expression under abiotic stresses
Source: BMC Genomics. 2022 Mar 7;23:190. doi: 10.1186/s12864-022-08416-9 (PMC8903722; doi:10.1186/s12864-022-08416-9)
Supplement: Supplementary file 3 — Additional file 3. [file 12864_2022_8416_MOESM3_ESM.docx]

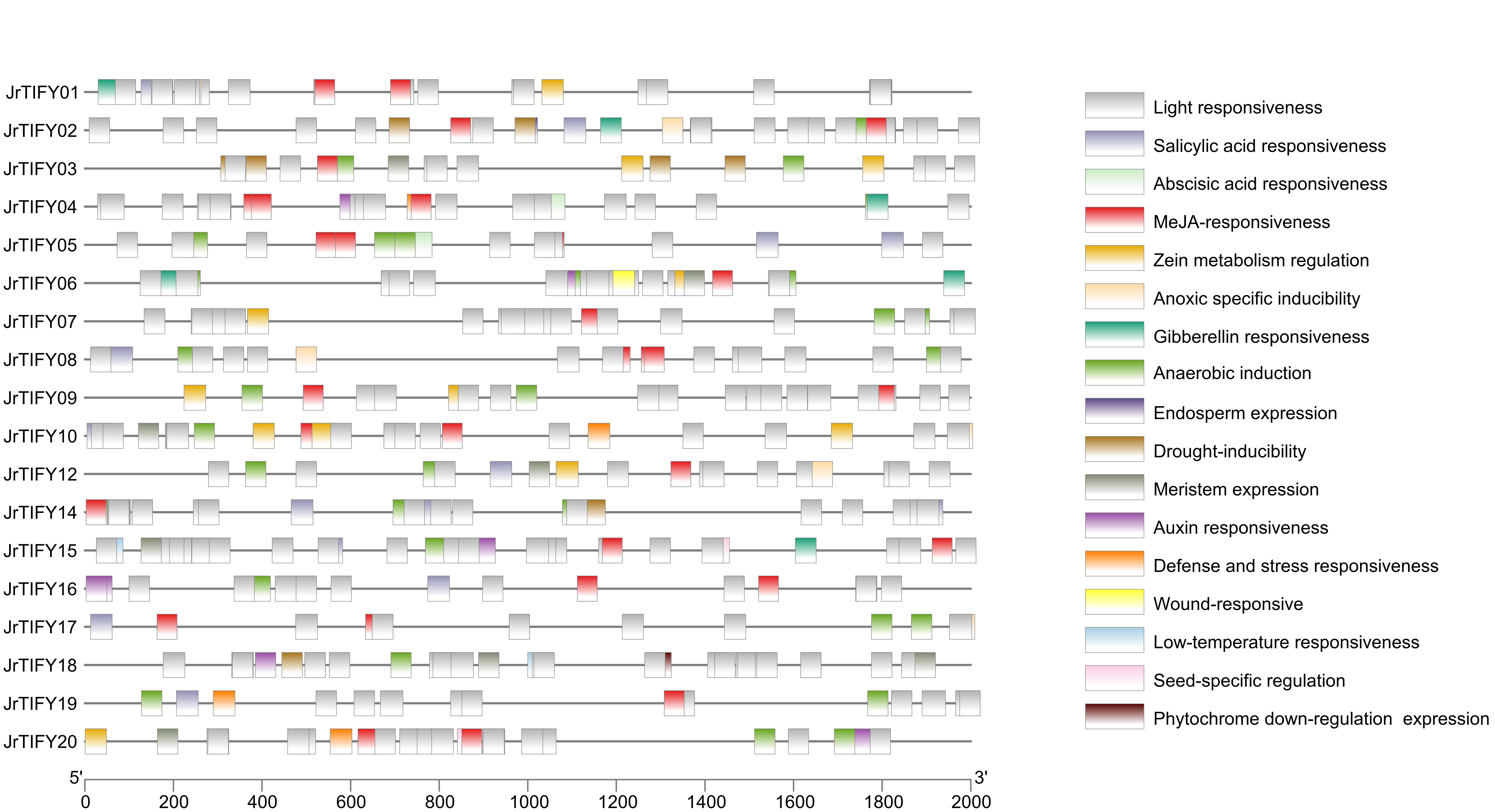


**Supplemental Figure 3. Promoter element of *JrTIFYs* genes about Fig. 6**. All promoter elements are shown in the diagram.
