# Supplementary material for: Identification of TIFY gene family in walnut and analysis of its expression under abiotic stresses
Source: BMC Genomics. 2022 Mar 7;23:190. doi: 10.1186/s12864-022-08416-9 (PMC8903722; doi:10.1186/s12864-022-08416-9)
Supplement: Supplementary file 4 — Additional file 4. [file 12864_2022_8416_MOESM4_ESM.docx]

| Locus | Forward primer 5'~3' | Reverse primer 5’~3’ | Size/bp |
| --- | --- | --- | --- |
| *JrTIFY01* | CTGGCATCATCTGGATCTATGACT | GGTTCTCCATCTCCACTTGCTT | 124 |
| *JrTIFY02* | GCTTCTGGCTGGGAATGTATCT | GAGCAAGGCTGTGTGTTTATGG | 132 |
| *JrTIFY03* | TAGTGGTGCCTCCGAGAGAAACT | TAATGTCTGCCTTGTTGGGATG | 200 |
| *JrTIFY04* | ACAAGGCAAGGGAGATCGTG | GGCGTTCTTGGGTGGTATTCT | 165 |
| *JrTIFY05* | TCCTTGTAACAATATCTCGCACATC | CTTTCTGTTTGCCTGACCCTCT | 121 |
| *JrTIFY06* | GCTCATGTTTTCGATGATGTCC | TTTCACCACCTGCCCTCAC | 121 |
| *JrTIFY07* | GGCTCCTGAATCAACCGTACTT | TTGCTTTCCTCGGGCTTG | 216 |
| *JrTIFY08* | TCGTCTTATTCCTCCTAGTACCACA | TCTGTTCGCCTGACCCTCTAC | 130 |
| *JrTIFY09* | CAAGTATTCCCACTGTTGCTTCC | TGAGCCTTTTCAGGGGTTATGT | 142 |
| *JrTIFY10* | CCCCGATAAGGTTCAAGCAGT | CTCCATCACACCACCCCTCT | 106 |
| *JrTIFY11* | GTCATCACCTTCGATAGCCTCA | AACATCAAGACGCAAAACTCCA | 146 |
| *JrTIFY12* | GTTGATTCCAGCAGTTCGATTG | AGGGGTTGAGTTGGTGGTTG | 104 |
| *JrTIFY14* | AACAGCCCAAGAGCCTTCC | GGTTCTACTTGCCCCTTCTCCT | 139 |
| *JrTIFY15* | CGGGGCAGGAAAATAAACAA | GCGAAGGGAAAGGGGAAA | 136 |
| *JrTIFY16* | CGAAGGAAACGGAAAGGTTG | CATGGGTTTAAGGTTCTGAGTGG | 127 |
| *JrTIFY19* | GAAGGGACATTGAACCCAAAAG | GGAGGAGACGCAAGTCAACC | 159 |
| 18S-rRNA | GGTCAATCTTCTCGTTCCCTT | TCGCATTTCGCTACGTTCTT | - |

**Table S1.** Gene-specific primers used in this study.
